# The effect of supernatant product of adipose tissue derived mesenchymal stem cells and density gradient centrifugation preparation methods on pregnancy in intrauterine insemination cycles: An RCT

**Published:** 2018-03

**Authors:** Hoda Fazaeli, Faezeh Davoodi, Naser Kalhor, Reza Tabatabaii Qomi

**Affiliations:** *Research Department, Highly Specialized Jihad Daneshgahi Infertility Treatment Center, Academic Center of Education, Culture and Research (ACECR), Qom, Iran.*

**Keywords:** SPAS, Preparation method, Pregnancy, IUI

## Abstract

**Background:**

One of the most important involved factors in pregnancy occurrence following intrauterine insemination (IUI) is semen sample preparation. Recently, supernatant product of adipose tissue derived mesenchymal stem cells (SPAS) method has been shown to improve semen parameters.

**Objective:**

To compare the effect of preparation methods in order to IUI, SPAS and density gradient centrifugation (DGC).

**Materials and Methods:**

This trial was done on 80 couples with male factor infertility who attend jihad daneshgahi infertility treatment center of Qom province, undergoing ovarian stimulation and IUI cycle. Various semen parameters including motility, count, DNA fragmentation and capacitation were evaluated before and after preparation. The effect of semen preparation methods and influence of various semen parameters on pregnancy occurrence were examined.

**Results:**

The overall clinical pregnancy rate was 17.5% per patient with no miscarriage. The pregnancy rate for DGC and SPAS were 5% (2 of 40) and 30% (12 of 40) respectively. Since there is no significant difference in improving motion parameters between two groups (except recovery of total number of motile spermatozoa), it seems that these parameters alone are not sufficient to predict IUI pregnancy outcome whereas in samples with >25 million motile spermatozoa in inseminate, there was a clear trend for a higher pregnancy rate for the sample processed using SPAS.

**Conclusion:**

Considering SPAS as a new and effective method leading to provide a combination of various improved semen parameters, is expected in near future.

## Introduction

One of the most important involved factors in pregnancy occurrence following intrauterine insemination (IUI) -the first line treatment for couples with unexplained and mild male factor sub fertility before in vitro fertilization- is semen sample preparation. Currently, density gradient centrifugation (DGC) is one of the routine methods in assisted reproduction technique laboratories. The efficiency of this method is completely depends on forming good interphases between medium gradients which needs special attention. After DGC, due to necessity of removing density medium, the obtained soft pellet containing qualitative sperms has to be washed. Thus, an additional centrifugation step is required. Repeated centrifugation, which may lead to significantly higher reactive oxygen species (ROS) production, is an important disadvantage of DGC (1). It is thought that membrane disruption is the responsible mechanism of ROS production by repeated centrifugation. These findings should be consider in assisted reproduction techniques, especially intracytoplasmic sperm injection, which requires spermatozoa with enhanced DNA integrity to avoid the inadvertent use of DNA- damaged spermatozoa (2, 3).

Moreover, there are some limitations in semen criteria to be selected for processing while severe oligoasthenozoospermia patients are not good candidates to undergo IUI due to low efficiency of common processing methods. So, it seems that there is still a lack of optimized semen preparation method which involves a wider range of patients while improves semen parameters.

During the past decade, different sources like the umbilical cord, umbilical cord blood, bone marrow (BM), adipose tissue (AT) and many other adult tissues are used to isolation of Mesenchymal Stem Cells (MSCs) as potential therapeutic strategies for a number of diseases (4), but AT and BM are the most widely used sources of MSC (5). It has been shown that human MSCs secret a variety of growth factors and cytokines such as interleukin (IL) 6, vascular endothelial growth factor, IL-8, granulocyte- colony stimulating factor, stem cell factor, insulin- like growth factor 1 (IGF-1), hepatocyte growth factor (HGF), interferon gamma, IL-15, IL-10, nerve growth factor, platelet- derived growth factor and basic fibroblast growth factor (6-8).

There are a large number of studies on stem cell derived secreted factors showing their repair ability in various conditions involved in tissue/ organ damage (9). Several studies used various methods to assess different cytokines in the conditioned medium, from the conventional ELISA assays (10, 11) to proteomic profiling methods (12, 13).

Recently, use of the supernatant product of adipose tissue derived mesenchymal stem cells (SPAS) method has been reported as a highly effective semen preparation technique improving sperm parameters including total and progressive motility as well as DNA fragmentation (14). The cause of selecting adipose tissue as MSCs source is higher secretion of some proteins involved in improving semen parameters such as basic fibroblast growth factor, interferon gamma and IGF-1 rather than bone marrow derived mesenchymal stem cells (15). Furthermore, there are some other biological advantages for adipose tissue derived mesenchymal stem cells (ATMSCs) in comparison to bone marrow derived mesenchymal stem cells including better proliferative capacity, higher immune-modulatory effects, easier accessibility to adipose source and subsequently higher number of recovered MSCs and noninvasive cell isolation of adipose tissue (14, 15). 

In this study, we compared the impact of two methods of sperm preparation, SPAS, and DGC on different sperm parameters including count, motion, morph, sperm capacitation and DNA fragmentation as well as pregnancy rate following IUI.

## Materials and methods

In this clinical trial, 80 couples with male factor infertility undergoing ovarian stimulation and IUI cycle, attending to infertility treatment center of ACECR (Academic Center of Education, Culture, and Research, Qom branch) from December 2015 until January 2017 participated. Couples who had one failed IUI with semen parameters including the concentration of at least 15×10^6^/ mL and 40-65% total motility without grade A (Rapidly progressive >20 μm/sec) were selected as the study group. Female partners with previous ovarian surgery, endocrine abnormalities (polycystic ovarian syndrome, thyroid disorders, hyperprolactinemia, hypogonadotropic hypo-gonadism), moderate/ severe endometriosis, and age >40 yr were considered as exclusion criteria. 

**Figure 1:**
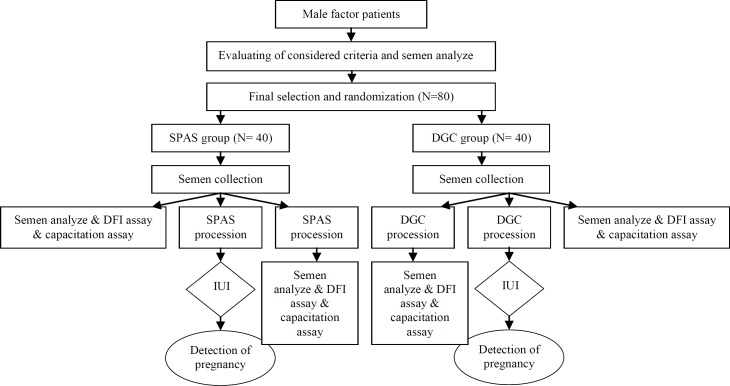
Flow chart of the study design.

Before and after preparation process, each specimen was evaluated for motility parameters and concentration, by use of computer- assisted semen analysis system. Morphology was evaluated in stained slides (using papanicolaou staining) according to criteria of Kruger (16). Furthermore, in order to assess the effect of DGC and SPAS on capacitation and DNA fragmentation, each specimen was evaluated before and after determined preparation procedure by use of chlortetracycline staining and sperm chromatin dispersion (SCD) test, respectively (Figure 1). 

**Evaluation of sperm DNA fragmentation**

SCD test (17) was performed using a Halosperm Kit (INDAS laboratories, Madrid, Spain) according to manufacturer's instructions. This test is based on the principle that normal spermatozoa with intact DNA strands can produce the characteristic big halo of DNA loops that is not generated from abnormal spermatozoa with fragmented DNA. Briefly, this involved adjusting the concentration of prepared and unprepared sperm with phosphate- buffered saline (PBS, sigma) to a concentration of 10 million/ mL and then adding 12.5 ml of each sample to 50 ml of melted agarose in a clean Eppendorf tube. 20 μL of the semen- agarose mix was pipetted onto slides precoated with agarose provided in the kit, and covered with a coverslip. The slides were placed on a cold plate in the refrigerator (4^o^C) for 5 min to allow the agarose to produce a microgel with the sperm cells embedded within. 

The coverslips were gently removed and the slides immersed horizontally in an acid solution which was previously prepared by mixing 80 μL of HCl from an Eppendorf tube in the kit with 10 mL of distilled water and incubated for 7 min. The slides were horizontally immersed in 10 mL of the lysing solution for 25 min. After 5 min washing in a tray with abundant distilled water, for 2 min the slides were dehydrated in increasing concentrations of ethanol (70%, 90%, 100%) and then air-dried. Each slide was examined under the light microscope at ×100 magnification and 200 sperm were scored.

**Evaluation of sperm capacitation**

Chlortetracycline (CTC, sigma) staining was used to evaluation of sperm capacitation. The method was as previously described (18, 19). Briefly, CTC solution (750 mM CTC, 5 mM cysteine in 130 mM NaCl and 20 mM Tris HCl) was prepared freshly, pH adjusted to 7.8 and stored at 4^o^C under dark condition. A mixture of sperm suspension and CTC solution in equal volumes (100 µl) was prepared in a Falcon tube at room temperature and then 2 µl of glutaraldehyde (sigma, 12.5% in 20 mM Tris-HCl, pH 7.4) was added to this mixture. 8 µl of solution was placed on a clean slide, to which 2 µl of 0.22 M 1, 4-diaza-bicyclo (2, 2, 2) octane (Sigma) dissolved in glycerol (Merk): PBS (9:1) were added to retard the fading of CTC fluorescence. 

The coverslip was placed on the sperm suspension and stored at 4^o^C in the dark. By use of phase- contrast and fluorescence microscopy (×1000) the slides were examined within 24 hr. Each sample was assessed twice and two hundred viable sperm were counted and classified into three patterns as follows (20): uniform bright fluorescence over the whole head (uncapacitated spermatozoa, pattern F), fluorescence- free band in the post acrosomal region (capacitated spermatozoa, pattern B), and dull fluorescence over the whole head except for a thin punctuate band of fluorescence along the equatorial section (acrosome reacted spermatozoa, pattern AR).

**Ovarian stimulation protocol**

IUI process was done in conjunction with controlled ovarian hyperstimulation (COH). COH was performed using Clomiphene citrate (CC, Iran hormone) and/ or gonadotropins like Follicle-stimulating hormone or human menopausal gonadotropin (Gonal-f® and Menogon, respectively, Germany). For CC stimulated cycles, between days 3 and 7, 100 mg of CC was given. For CC plus gonadotropin stimulation, 100 mg of CC was given similarly with 150 U/day of gonadotropins added by day 9. Only on day 3 with 75-150 U/day the stimulation with gonadotropins was started. The dosage of gonadotropins on subsequent days of COH was desighned according to follicular maturation, which was monitored by serial transvaginal ultrasonography. When the diameter of the leading follicle(s) became ≥18 mm, patients received 10,000 U of Human chorionic gonadotropin (hCG, PD preg). 

**Semen preparation protocol**

After a mandatory omission of sexual activity for 2-7 days, all specimens were collected. Semen samples were placed in a 37^o^C incubator for 30 min to allow liquefaction to take place. Randomization method for sperm preparation for IUI was done according to the sample number starting with number 1. The sample was assigned to be prepared with SPAS followed by DGC the following number.

**DGC technique**

DGC procedure was done as described before, but with a little modifications (3). Briefly, adding Hams F10 supplemented with 2.5% Human Serum Albumin (HSA, Gibco), density gradient media (Allgrad, Life global) was prepared in 40 and 80% concentrations. The density gradient was prepared by layering 2 ml of 80% medium under 2ml of 40% medium in a conical centrifuge tube. 2 ml of the liquefied semen sample was placed over the upper layer (40%) by use of a sterile pipette, and then it was centrifuged at 300 g for 15 min. At the end of the centrifugation, most of the supernatant was gently removed and the pellet was placed into a new, clean tube. Then pellet was well resuspended in 5 ml of washing medium (Hams F10+2.5% HSA) in order to remove the density gradient medium. After centrifugation, the supernatant was removed and 5 ml of new medium was added. The centrifugation was repeated again and the final pellet was resuspended in the sterile medium.

**SPAS technique**

Isolation of ATMSCs was carried out as described previously by Fazaeli *et al* (14). ATMSCs in 3^rd^-9^th^ passage were used for the experiment. At approximately 80% confluency (8×10^5^ cell/cm^2^), the medium of ATMSCs was refreshed and cultured for an additional 48 hr. SPAS was collected and filtered by a 0.22 um membrane. It was stored at -4^o^C or -80^o^C before use in the following experiment. Before storing, a sample of collected SPAS was evaluated by microbial culture to ensure lack of any contamination. 

In the day of IUI, defreezing of SPAS was done in a water bath at 37^o^C. The total sample was divided in to 1 mL volumes in test tubes and 1.5-2 mL of SPAS was slowly added to the tube. Then the tubes were placed in the 37^o^C incubator at an angel of 45 degrees for 40 min (14). Using a sterile pipette the supernatant was gently collected and finally, it was centrifuged with 5 mL of Hams F10+ 2.5% HSA at 2500g for 5 min. After centrifugation, most of the supernatant was removed and 0.5-0.7 ml remained medium+ pellet was used for IUI.

**Detection of Mycoplasma contamination using polymerase chain reaction (PCR)**

Although Mycoplasmas do not cause visible damages in cells, they have considerable effects on cell metabolism and growth in the culture medium, protein synthesis, secretion of cytokines, and even may cause damage to DNA and RNA. Moreover, because of impossibility in visual or microscopic identification of Mycoplasma contamination in most cases, molecular methods based on PCR are preferred due to their innate features. We assessed SPAS by this method to be sure about lack of contamination with most identified mycoplasma species cell cultures like Mycoplasma fermentans, Mycoplasma hyorinis, Mycoplasma arginini, Mycoplasma orale and Achoplasma laidlawi. PCR was done according to previously described method (21). Briefly, DNA extraction was done using Exgene TM cell, clinic, blood kit (gene All) and after PCR, gel electrophoresis of PCR product was done.

**Insemination protocol**

Intrauterine insemination was performed 36 h after injection of hCG. The IUI was performed using an IUI catheter (Techwin Medical CO, Iran) with a final volume of 0.5-0.7 ml processed semen. Patients received daily Intramuscular administration of 50 mg progesterone (Iran hormone, Iran) suppositories for luteal phase support starting the day of IUI.

**Detection of pregnancy**

Pregnancy testing was performed through determining the quantitative serum β-hCG level at 14th day of IUI procedure, hCG >20 mIU/ mL were considered as biochemical pregnancy. The confirmation of a clinical pregnancy was done by the testing the presence of a gestational sac on transvaginal ultrasonography or by histological examination conception products in patients who were aborted.

**Ethical consideration**

This study was undertaken upon approval by the ethics committee of Islamic Azad University, Tehran Medical Branch, Tehran, Iran (IR.IAU.TMU.REC.1395.373). Written informed consent was obtained from all participants before any action. 

**Statistical analysis**

All data distributions were tested for skewness and kurtosis using Statistics Package for the Social Sciences software, version 11.5 (SPSS. Inc., Chica, IL, USA). All the variables showed normal distributions. Statistical significance was determined using Student,s *t*-test, Paired sample test, and Chi squared test. p-value of ≤0.05 was considered significant.

## Results

Half of the participations underwent IUI with DGC while for the other half, SPAS was used as the preparation method. 

**Obtained data of female partner related factors**

The mean age (±SD) for the women was 29.33±4.325 yr. Overall, the women had a mean body mass index of 25.5±3.538, a mean day-3 follicle-stimulating hormone of 6.946±2.304 IU/L, LH of 7.025±3.792 IU/L, estradiol of 50.54±35.11 pg/mL, and 2.84±1.373 follicles on the day of hCG administration. There were no differences among the groups for noted criteria above (Table I). 

**Comparison of sperm motion and morphologic parameters before and after processing**

Various semen parameters including those before and after preparation are shown in table II. As shown parameters related to sperm motion and normal morphology were not statistically different between two groups except the percentage of recovery of motility which was more favorable after SPAS (p=0.018). However, compared with the original semen, in both groups, an improvement of sperm motion and normal morphology parameters was observed after sperm preparation, while overall sperm concentration was reduced (p≤0.001). 

**Comparison of sperm capacitation before and after processing**

Due to assessing the efficiency of applied preparation methods in capacitating spermatozoa, each sample was evaluated by CTC staining before and after the process. As it is shown in figure 1, there was not only a significant increase in percentage of capacitated spermatozoa in both groups after processing but this parameter was more favorable after SPAS rather than DGC (p≤0.001), (capacitated percentage: 21.675±1.817 and 21.32±1.67 for DGC and SPAS controls respectively and 55.4±6.34 and 58.45±6.34 after DGC and SPAS processing respectively). 

**Comparison of sperm DNA fragmentation before and after processing**

**Figure 2:**
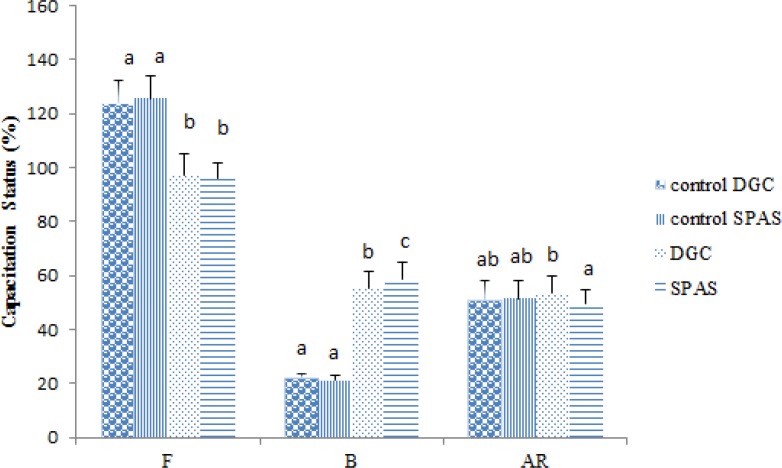
Evaluation of capacitation status of spermatozoa prepared using SPAS and DGC methods by CTC staining. F: intact none capacitated, B: capacitated, AR: acrosome reacted spermatozoa.

In terms of sperm DNA fragmentation, as it is shown in figure 2 the obtained results revealed a significant decrease of this parameter after preparation in both groups (41.55±6.093 vs. 23.68±5.337 for SPAS group and 41.73±5.44 vs. 26.53±4.529 for DGC group). Moreover, SPAS method acted more effectively in decreasing DNA fragmentation than DGC method (p=0.012). Overall, 14 cycles resulted in pregnancy after IUI. Thus, the overall clinical pregnancy rate was 17.5%. The pregnancy rate for DGC was 5% (2 of 40) and the rate for SPAS was 30% (12 of 40).

**Comparison of pregnancy rate before and after processing**

If there was any difference between two groups in case of pregnancy rate, total motile sperm inseminated as one of the most common described effective parameter in pregnancy occurrence of IUI was assessed (Table III). When <25 million motile spermatozoa were inseminated, the number of pregnancy in both groups was not enough to lead to a valid conclusion. However, for SPAS group, there was a clear tendency for higher pregnancy number. 

When 25-35 million motile sperm were applied to insemination, the total rate of clinical pregnancy showed statistically significant improvement (6.66% for the DGC vs. 39% for the SPAS). At 35-45 million motile spermatozoa clinical pregnancy rate of SPAS was still significantly better than DGC group (7.15% for the DGC vs. 44.4% for the SPAS) while by inseminating more than 45 million motile spermatozoa no significant difference was observed between 2 groups which may be the result of low number of performed cycles with this inseminated motile count. In this study, all the biochemical pregnancies developed to clinical pregnancies and no abortion was reported.

**Table I:** Female partner related factors in SPAS and DGC groups

**Female partner parameters**	**SPAS**	**DGC**
Age	29.3 ± 3.891^a^	29.35 ± 4.769 ^a^
BMI	24.754 ± 2.49 ^a^	25.647 ± 4.329 ^a^
LH	7.193 ± 2.946 ^a^	6.857 ± 4.514 ^a^
FSH	6.677 ± 1.354 ^a^	7.214 ± 2.96 ^a^
Estradiol	48.762 ± 18.762 ^a^	52.32 ± 46.247 ^a^
Number of follicles	2.8 ± 1.224^a^	2.88 ± 1.522 ^a^

Data presented as mean ± SD. Independent sample test.

BMI: Body mass index

LH: Luteinizing hormone

FSH: Follicle-stimulating hormone

SPAS: Supernatant product of adipose tissue derived mesenchymal stem cells

DGC: Density gradient centrifugation

a ; p≤0.05 Comparison between SPAS and DGC groups

**Table II:** Sperm parameters before and after preparation

		**SPAS**	**DGC**
Pre- preparation			
	Concentration (×10^6 ^ml)	104.63 ± 18.411^m, ^^a^	99.13 ± 28.123 ^a^
	Total motility (%)	53.88 ± 11.294 ^m, ^^a^	58.25 ± 7.97 ^a^
	Progressive motility	20.88 ± 11.373 ^m, ^^a^	23.13 ± 6.474 ^a^
	Normal morphology	3.85 ± 0.622	3.55 ± 0.512^a^
Post- preparation			
	Concentration (×10^6 ^ml)	82.5 ± 17.65 ^m^	81.88 ± 26.763
	Total motility (%)	69.63 ± 11.174 ^m^	70.13 ± 7.965
	Progressive motility	42 ± 16.164 ^m^	47.13 ± 12.704
	Normal morphology	5.45 ± 0.552^m^	5.33 ± 0.572
	Inseminated motile count	28.91 ± 8.341 ^m^	29.14 ± 10.8
	Recovery rate of count (%)	79.19 ± 11.728 ^m^	82.2 ± 14.817
	Recovery rate of motility (%)	129.23 ± 27.698^n^	120.39 ± 11.238

Data presented as mean ± SD. Independent sample test.

SPAS: Supernatant product of adipose tissue derived mesenchymal stem cells

DGC: Density gradient centrifugation

a ; p≤0.05 Comparison between pre- and post- preparation; m; p>0.05, n; p≤0.05 Comparison between SPAS and DGC methods;

**Table III:** Clinical pregnancy rate according to preparation method and number of inseminated motile spermatozoa

**Inseminated motile count**	**Cycles**	**Clinical pregnancies**		**p-value**
		**DGC**	**SPAS**	
<15 × 10^6^	5	0 (0)	0 (0)	1
15- 25 × 10^6^	17	0 (0)	1 (10)	0.88
25.1- 35 × 10^6^	33	1 (6.66)	7 (39)	0.032
35.1 - 45 × 10^6^	23	1 (7.15)	4 (44.44)	0.034
≥45.1 × 10^6^	2	0 (0)	0 (0)	1

All data presented as n (%).

Chi squared test

SPAS: Supernatant product of adipose tissue derived mesenchymal stem cells

DGC: Density gradient centrifugation

**Identical letters represent lack of significant difference while non- identical letters show significant difference (p ≤0.05)**

**Figure 3:**
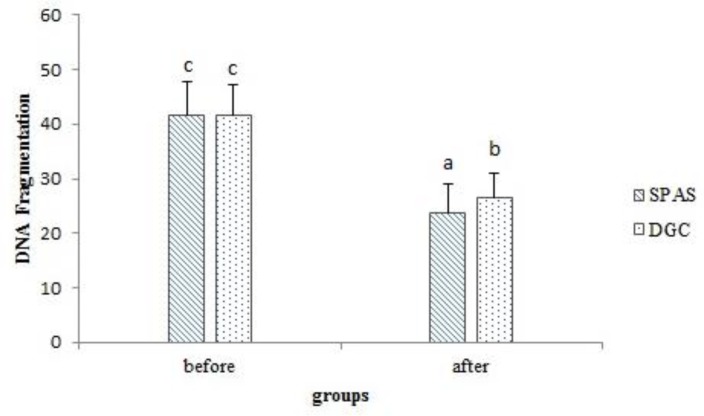
Evaluation of sperm DNA fragmentation before and after semen processing using SPAS and DGC methods.

**Identical letters represent lack of significant difference while non- identical letters show significant difference (p ≤0.05).**

## Discussion

In this study, SPAS and DGC methods were chosen to process semen before performing IUI in order to assess these methods impact on both semen parameters and the pregnancy rate. According to the obtained data, whether DGC or SPAS was applied, motion parameters (total and progressive) and the percentage of recovered normal morphology were enhanced significantly (p≤0.05). However, no statistically significant difference were found between two groups in these parameters (except for the recovery of total motile spermatozoa which was significantly higher in SPAS group) showing their equal efficiency motion and morphologic parameters. 

Considering the nature of DGC method which is based on the isolation of live sperms from dead ones according to their different density, improvement in total and progressive motility is expected while a large number of previous studies as well confirm it (22). The rich content in growth factors of SPAS may be responsible for the mentioned parameters improvement. This result is in agreement with the findings of who found that this preparation method helped the isolation of spermatozoa (14). There are a number of studies investigating the growth factors role in spermatogenesis as following: In 2012 it is reported that development, maturation, and motility of the spermatozoa will be affected by variations in IGF-1 levels of seminal plasma (8). Due to the presence of large amounts of HGF in the distal part of the epididymis and the acquisition of motility by immotile spermatozoa cultured with HGF, Naz and his colleagues suggest that this growth factor can induce sperm motility (23). 

Localization of FGF2 in the female reproductive tract is confirmed, whereas its secretion by the oviduct epithelial cells, the oocyte and the cumulus oophorus cells has been reported (24, 25). Besides, the expression of fibroblast growth factor receptor 1, 2, 3 and 4 in the human testis and sperm and their limited localization to acrosomal region and flagellum is demonstrated. Based on the findings that showed the exposure to FGF2 leads to these receptors phosphorylation and activation of intracellular pathways which are involved in the maintenance of sperm motility, capacitation, acrosomal exocytosis and survival, the functionality of them were proven (26, 27) while in a study conducted in 2015 an increase in the percentage of motile cells and enhancement of sperm kinematics has been reported (28).

We found a clear trend for a higher pregnancy rate in samples processed using SPAS which indicates motion and morphologic parameters and the number of spermatozoa in the inseminate may not regarded as determinants of pregnancy outcome which is in contrast with previous studies (29, 30). It means that when the efficacy of preparation methods in improving sperm motion parameters is alike, these parameters are not sufficient to predict pregnancy outcome of IUI anymore. Similarly, as it is shown in table III, when the total number of motile spermatozoa inseminated exceeded 25 million, the preferred method of sperm preparation should be SPAS. It urges us to pay more attention to other sperm parameters influenced by preparation methods such as capacitation and DNA fragmentation.

In this study, we found that although processing with both methods leads to improve capacitation percent, still the efficiency of SPAS was significantly more than DGC (p≤0.05). 

In order to explain the mechanism of SPAS method to elevate capacitation, we should focus on its content and procedure. Furuya and his colleagues found that activating the tyrosine kinase of EGFR on spermatozoa is the mechanism by which EGF could stimulate human sperm capacitation (30). Moreover, in a study on porcine spermatozoa assessing possible effects of centrifugation on calcium influx dynamics under capacitation condition, it was shown that DGC either through percoll or single layer source, gradually decreased spermatozoa response to the capacitation stimulus bicarbonate (31). Then, the ability of DGC as a dependent method to duration and centrifugation force expected to be lower than SPAS method with least centrifugation levels.

Our findings of significant decline of sperm DNA fragmentation after processing is in agreement with Lamirande and his colleagues (32). Using different stains and tests, they evaluated some of capacitation and acrosome reaction derived changes that may occur in human sperm head (32). Their obtained results were related to physiological cell activation, since they originate from the normal prerequisites for fertilization, purified motile spermatozoa undergoing capacitation and acrosome reaction. They also suggested that possible mechanisms of sperm chromatin remodeling are not yet known, but hypothesized that maybe by reduction of disulfide bridges, changes are initiated during capacitation, which would decrease chromatin compaction and allow reorientation of histones. 

Our obtained data represent the different ability of SPAS and DGC to separate spermatozoa possessing nuclear anomalies from those that are normal. Also due to higher capacitation induced by SPAS, a significant decrease of DNA fragmentation is not unexpected in the light of the above considerations. Beside, a study in human spermatozoa suggests that during DGC, transition metals in density gradient media cause the spermatozoa to face oxidative damage that leads to DNA damage (33).

Due to more capability of capacitated sperm in fertilization either by the use of intrauterine insemination or the other kinds of in vitro fertilization technologies, insemination of semen samples prepared with SPAS methods yield to higher pregnancy rate as it is shown in this study. 

## Conclusion

Consideration of SPAS as a new effective clinical method, which mimics in vivo environment including growth factors causing a combination of different sets of improved semen parameters, is expected in the near future.

## Note

***Registration ID in IRCT: IRCT2017021532583N1***
